# A bacteriophage journey at the European Medicines Agency

**DOI:** 10.1093/femsle/fnv225

**Published:** 2015-12-11

**Authors:** Laurent Debarbieux, Jean-Paul Pirnay, Gilbert Verbeken, Daniel De Vos, Maia Merabishvili, Isabelle Huys, Olivier Patey, Dirk Schoonjans, Mario Vaneechoutte, Martin Zizi, Christine Rohde

**Affiliations:** P.H.A.G.E., Queen Astrid Military Hospital, Lab. for Molecular and Cellular Technology (LabMCT) Bruynstraat 1, 1120 Brussels, Belgium

## Abstract

The seriously and globally increasing bacterial multi-drug resistance calls out on concerted counteractive measures: international health authorities give consideration to the therapeutical use of bacteriophage therapy.

## BACKGROUND

On 8 June, 2015, London, a workshop on bacteriophage therapy was organized by the European Medicines Agency (EMA). About 60 delegates from various stakeholders (participants belonged to public bodies such as WHO, ECDC, WAAAR, national health agencies from different European countries, politicians, journalists, clinicians, researchers, including six members of P.H.A.G.E., as well as private companies.) were convened to participate, with the workshop broadcast on the EMA website. Why did EMA organize a workshop on bacteriophage therapy? The answer is the emergency to find solutions to overcome the global antibiotic crisis and the unfortunately corresponding lack of significant interest in bacteriophage therapy. The past two decades, public health agencies reported on the dramatic increase of drug-resistant pathogens, a worrisome situation leading the World Health Organization (WHO) to declare a new ‘preantibiotic era’ in its 2014 surveillance report (http://www.who.int). In the USA, based on data collected in 2009, over 20 000 annual deaths were ascribed to the lack of effective antibiotics. Comparably in Europe, we estimate that more than 100 000 people worldwide die annually. The situation gets worse: a recent study mentions that in France about 13 000 people died in 2012 (http://www.invs.sante.fr). Worldwide, the estimation is rising to 700 000 deaths annually, and therefore G7 Health Ministries recently called for a global ‘One Health’ approach (https://www.g7germany.de). Future predictions are alarming, with 10 million people estimated to die from resistant microorganisms in 2050 if nothing changes (Carlet 2015).

## FROM FIXED ANTIBIOTICS TO FLEXIBLE BACTERIOPHAGES?

Bacteriophage therapy is nearly 100 years old and was first introduced in France and Belgium a decade before the discovery of penicillin by A. Fleming. After an initial worldwide interest (including from the pharmaceutical industry), bacteriophage therapy was abandoned in the Western World in the 1940s due to the advent of more convenient broad spectrum antibiotics and the unreliability of the early bacteriophage preparations. Meanwhile, in the former Soviet Union bacteriophage therapy was further developed. Over the past century, thousands if not millions of patients have been treated with bacteriophages and today two phage therapy centers, in Poland and in Georgia, are still operational. Interestingly, in Poland—a European Union (EU) Member State—bacteriophage therapy is currently used to treat antibiotic-resistant bacterial infections. Such treatment is performed under the umbrella of Article 35 of the Declaration of Helsinki (reading ‘treatment of a patient, where proven interventions do not exist’) and is done with the consent of the patient (Międzybrodzki *et al*. 2012). With an increasing number of patients who could benefit from bacteriophage therapy, the current situation, in which some EU patients have access to an alternative treatment while others do not, is not sustainable and should be dealt with on an EU level. It is certainly not the first time that medicine has faced health inequalities that need to be solved at a political level. Examples are currently authorized drugs such as antiretroviral therapy for HIV or vaccines for avian influenza patients and more recently experimental treatment for Ebola virus, for which access and availability were worked out politically to deal with major public health issues.

Bacteriophage therapy is facing several challenges. First, due to an insufficient interest from established Western pharmaceutical companies, no commercial bacteriophage preparations for human use are currently available in the EU or in the United States. Second competent medical authorities had not anticipated that bacteriophage-based products would benefit from, or even need, adapted requirements for production, quality and safety. This has led to a substantial delay in starting the first multi-center phase II clinical trial funded by the European Commission (www.phagoburn.eu). Third, the evolving nature of bacteriophages, allowing them to infect evolving bacteria, as well as the ingenious therapeutic feature of being self-replicating and self-limiting at the site of infection (one main advantage of bacteriophages over antibiotics), makes them very different from any other known pharmaceutical product. Finally, the ability to select bacteriophages that can specifically target the infecting bacteria in individual patients is opening the door for sustainable tailor-made approaches, which are outside of the range of the current antimicrobials. Bacteriophage therapy is thus unique, and therefore would benefit from a dedicated framework, which in many ways should differ from the current medicinal product (antibiotics) development and marketing pathways.

The EMA clearly stipulated during this workshop that none of the regulations currently fit bacteriophage therapy adequately (http://www.ema.europa.eu). Nevertheless, the French, Belgian and Swiss competent authorities for medicines approved the phage cocktails that will be tested in the PhagoBurn clinical trial. Since this study benefits from EU funding, the authorities were inclined to give ground to facilitate the approval of the PhagoBurn phage cocktails. However, this step forward does not offer a sustainable solution for future bacteriophage therapy approaches. Clearly, two main (connected) barriers need to be overcome. First, the regulatory pathway should be adapted through the establishment of dedicated definitions and rules. These rules are necessary to allow the field to tackle the second barrier, which is the lack of public and private funding. As mentioned in an earlier publication, both ‘prêt à porter’ (for cohorts of patients) and ‘sur mesure’ (for individuals) approaches can live side by side, as they will complement each other (Pirnay *et al*. 2011). While the ‘prêt à porter’ approach may require some long-term investments for production, the ‘sur mesure’ approach could be more rapidly implemented. Under the initiative of colleagues from the Queen Astrid Military Hospital in Brussels, an article on the preparation of a bacteriophage solution, within the hospital laboratory, was published in 2009, and recently broader consensus elaborated by 32 experts in the field on the quality and safety requirements for bacteriophage products was published (Merabishvili *et al*. 2009; Pirnay *et al*. 2015).

Now it is time that political decision and public investment make a difference. The debate is no longer about the pro and cons of phage therapy, but rather about how we can move forward for patients to benefit from this therapy. We are proposing to set up dedicated public structures, National Reference Centers (NRCs) for bacteriophage therapy. These NRCs will pilot these treatments and put in place production of hospital-based bacteriophage solutions, and application protocols that will ensure adequate product quality, patient safety and monitoring of treatment efficacy. Even though bacteriophage therapy is rather straightforward, matching bacteriophage to bacteria, advising clinicians and keeping records of all treatments is crucial as this information will be useful in setting up subsequent large clinical trials, which could be sponsored by pharmaceutical companies. These NRCs should host or have access to a research team, for isolating specific bacteriophages, as well as a bacteriophage bank for providing long-term storage. From well-documented clinical cases from the NRCs, relevant scientific questions will emerge to dictate the paths that urgently need to be explored and studied, instead of expecting basic science to run almost unlimited investigations since many molecular aspects of phage therapy are still not understood. If we had waited for immunology to be fully understood at the molecular level before the use of vaccines, most of us would not be reading this paper! A similar approach has been recently proposed in the USA (Kutter *et al*. 2015) but *in fine* none of these proposals will ever be close to reality if regulations are not adapted to bacteriophages, and not the other way around.

The EMA workshop ended with the recommendation to promote additional clinical trials to further document how bacteriophages should best be applied in clinical practice. However, without speedy adaptation of the medicinal product regulation to support sustainable development of bacteriophage therapy, this process will be unacceptably lengthy as established Western pharmaceutical companies will not invest in the absence of adequate rules and intellectual property protection. Meanwhile, the list of patients requiring antimicrobial treatments will continuously increase (Witzenrath 2015). The time you read this, 10 persons died because no effective antibacterial treatment was available. Which threshold of deaths will be reached before bacteriophages are allowed to be used to help save lives?
